# Integrative Histology-Genomic Analysis Predicts Hepatocellular Carcinoma Prognosis Using Deep Learning

**DOI:** 10.3390/genes13101770

**Published:** 2022-09-30

**Authors:** Jiaxin Hou, Xiaoqi Jia, Yaoqin Xie, Wenjian Qin

**Affiliations:** 1 Shenzhen Institute of Advanced Technology, Chinese Academy of Sciences, Shenzhen 518055, China; 2 Shenzhen College of Advanced Technology, University of Chinese Academy of Sciences, Shenzhen 518055, China; 3 School of Computer Science and Engineering, Northeastern University, Shenyang 110819, China

**Keywords:** multi-modality, hepatocellular carcinoma, prognosis, deep learning

## Abstract

Cancer prognosis analysis is of essential interest in clinical practice. In order to explore the prognostic power of computational histopathology and genomics, this paper constructs a multi-modality prognostic model for survival prediction. We collected 346 patients diagnosed with hepatocellular carcinoma (HCC) from The Cancer Genome Atlas (TCGA), each patient has 1–3 whole slide images (WSIs) and an mRNA expression file. WSIs were processed by a multi-instance deep learning model to obtain the patient-level survival risk scores; mRNA expression data were processed by weighted gene co-expression network analysis (WGCNA), and the top hub genes of each module were extracted as risk factors. Information from two modalities was integrated by Cox proportional hazard model to predict patient outcomes. The overall survival predictions of the multi-modality model (Concordance index (C-index): 0.746, 95% confidence interval (CI): ±0.077) outperformed these based on histopathology risk score or hub genes, respectively. Furthermore, in the prediction of 1-year and 3-year survival, the area under curve of the model achieved 0.816 and 0.810. In conclusion, this paper provides an effective workflow for multi-modality prognosis of HCC, the integration of histopathology and genomic information has the potential to assist clinical prognosis management.

## 1. Introduction

Liver cancer is one of the most commonly diagnosed cancer with the sixth incidence rate and the third mortality rate all over the world in 2020 [1]. In this study, we focus on hepatocellular carcinoma (HCC), which is the most prevalent subtype of primary liver cancer. The poor prognosis of liver cancer whose general 5-year survival rate is just 20%, has attracted the attention of doctors and researchers [2].

Prognosis prediction is essential in clinical practice which models time-to-event outcomes. It is useful for strategy optimization and effect evaluation of treatment [3]. However, because of the highly heterogeneous of cancer, the prognosis of patients is usually affected by many factors. It often requires various examinations to collect multi-modality data for a comprehensive assessment. In addition to the patient’s own physical conditions, the doctor’s experience will also affect the accuracy of the prognosis. Unlike other diagnoses, a definite diagnostic standard for an exact survival time prediction is difficult to establish. Accurate prognosis prediction is still a challenge. With the development of computer science, it is a new choice to use computational methods to predict the prognosis of cancer patients [4].

Pathological diagnosis is the gold standard of cancer diagnosis that is a necessary basis for cancer classification, grading, and staging. In clinical practice, pathologists usually observe pathology sections under a microscope and make a diagnosis according to the observed morphological features of tumor cells and the histological structure of the tumor microenvironment.

With the popularity of digital scanning of pathological images, algorithms based on the whole slide images (WSIs) have developed rapidly in recent years, and a large number of papers on cancer diagnosis and prognosis based on digital pathological images have been published, such as diagnosis algorithm based on weakly-supervised [5], tumor mutational burden prediction algorithm based on multiscale learning [6], segmentation and classification algorithm of colonoscopy WSI [7]. H. Bhargava [8] et al. extracted features from WSIs, which can be used as computational signatures for prostate cancer prognosis. This research further explored the relationship between tumor biomarkers and stromal morphological characteristics. X. Zhu [9] et al. proposed a WSIs survival prediction framework based on deep learning and designed the first loss function for convolutional neural network prognosis. However, survival prediction just based on WSIs is usually inaccurate and hardly meets the clinical-grade application.

There is an assumption that multiple modality data can provide information from different perspectives leading to a more accurate prediction of patient outcomes. There is also some research explored in the field of multi-modality survival prediction. In these research, the commonly used modalities include histopathology, radiomics, clinical characteristics, genomics, and other high-throughput molecular data. Genomics can be further divided into multiple modalities, such as mRNA expression sequence, methylation, and mutation. Similarly, radiomics include computed tomography and nuclear magnetic resonance imaging of various sequences. The purpose of multi-modality studies [10,11] is usually to build an automatic diagnosis or prognosis model, extract features from each modality, integrate features, and use statistical models, machine learning, or deep learning methods to make the prediction. In most multi-modality prognosis studies, researchers choose the combination of modalities in genomics [12] or other modalities with genomics [13]. In a pan-cancer study [14], a prognosis model was developed with six modalities based on deep learning. For some cancer types, the concordance index (C-index) approached the optimal score of 1.0. This study also proved gene expression data contributed the most to the prognosis model through controlled experiments. M. Fan [15] et al. identified heterogeneous breast cancer subtypes by radiomics and genomics signatures. The subtypes were demonstrated to have different biological functions and survival outcomes. J. Cheng [16] et al. integrated genomic features and morphological features extracted from WSIs to build a survival prediction model for clear cell renal cell carcinoma and explored the relationship between the two modalities. These studies demonstrate the effectiveness of genomic data in prognosis prediction.

Throughout the existing studies, there are fewer studies on multi-modality survival prediction of liver cancer than other cancer. In addition, most of the existing studies use machine learning methods to extract image features by hand. Therefore, the information extracted by deep learning methods still needs further exploration to find a new reliable biomarker.

In this paper, we designed a workflow of multi-modality survival analysis for HCC and built a prognosis prediction model. We collected multi-modality data from the public dataset The Cancer Genomic Atlas (TCGA) [17] and cBioportal, including WSIs and mRNA expression profiles. In the workflow, a state-of-the-art deep learning model was used for WSIs to calculate patient risk scores, and weighted gene co-expression network analysis (WGCNA) was used to summarize the gene expression information. Then, a multi-modality integrated prognostic model for HCC was established based on Cox proportional hazards model. The model performance demonstrated by multiple evaluation metrics. Decision curve analysis (DCA) was also used to evaluate the clinical value of the integrated model.

## 2. Materials and Methods

### 2.1. Patient Cohorts

In project TCGA liver hepatocellular carcinoma collection (LIHC) on cBioportal, there are 369 patients diagnosed with HCC. For genomic, we excluded 3 patients without mRNA expression files and downloaded files of the other 366 patients. These genetic data are log-transformed mRNA expression z-scores that compared to the expression distribution of all samples, and each patient has 20,531 gene measurements. Then, 6 patients with missing follow-up were excluded.

For histopathology, we collected 357 WSIs of 349 patients in project TCGA-LIHC on TCGA. Each patient has 1–3 WSIs. All the images were obtained by scanning hematoxylin-eosin-stained paraffin-embedded sections and stored with magnifications from ×5 to ×40. We matched the samples from TCGA with those from cBioportal and excluded the patients who just have single modality data.

**Figure 1:**
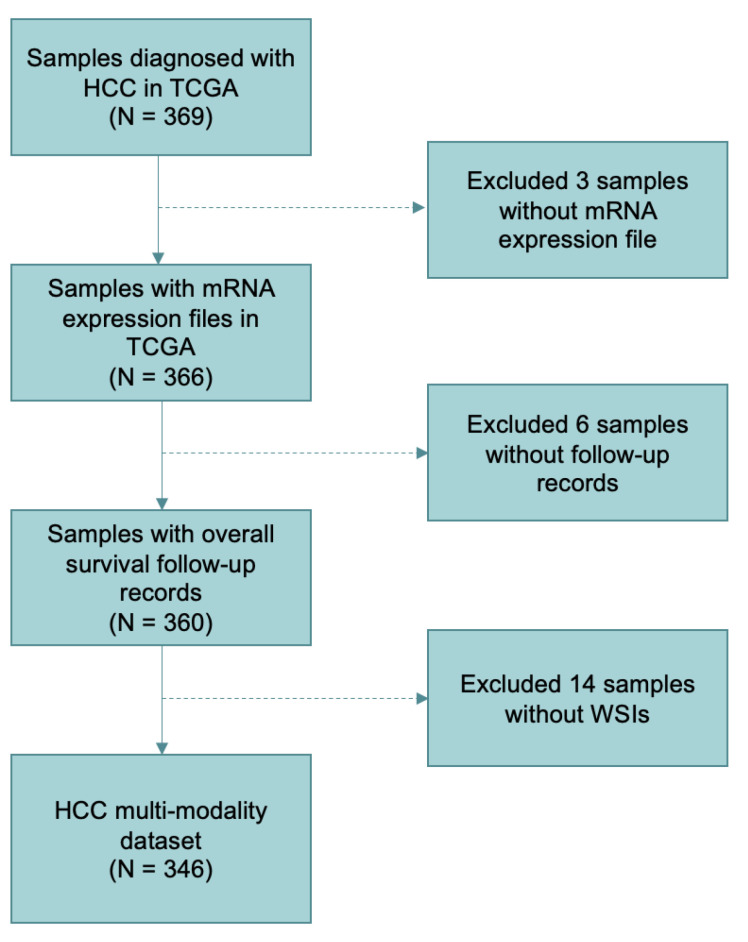
Sample screening and data organization flowchart in the study. A total of 346 patients were included in the datasets according to the selection criteria.

After screening, 346 patients with WSIs, mRNA expression files, and clinical survival information were retained totally. The details of including and excluding steps are shown in Figure 1.

### 2.2. Data Analysis and Integration Workflow

**Figure 2:**
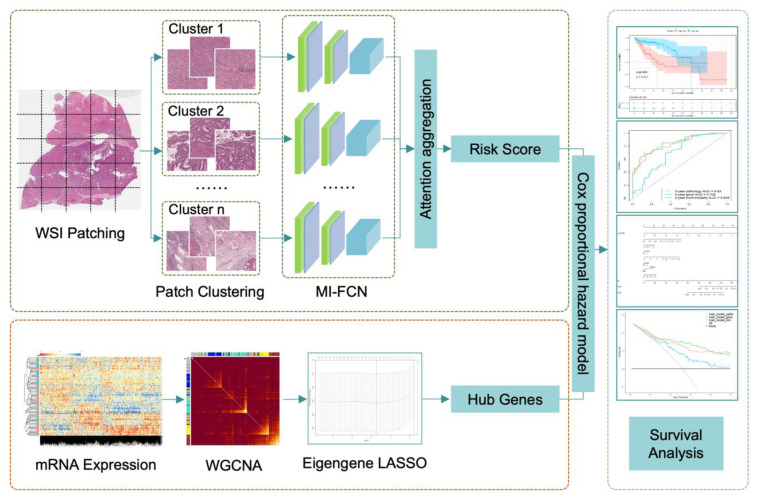
The workflow chart of key steps in our study. In the part of histopathology image processing, patches cut from one patient were clustered into 10 categories. Patches of each category were input into the multi-instance fully convolutional network (MI-FCN), and then, the outputs were aggregated with attention mechanism to get a risk score of the patient. In the part of mRNA-seq processing, eigengenes were obtained by weighted gene co-expression network analysis (WGCNA). Then, modules were selected by the least absolute shrinkage and selection operator (LASSO) based on eigengenes. Top hub genes of the retained modules were extracted as risk factors. In the last part, the deep learning risk score and hub genes were integrated together by Cox proportional hazard model.

The workflow consists of three parts: histopathology images processing, mRNA-seq analysis by WGCNA, and multi-modality survival analysis. The key steps are shown in Figure 2. The three parts will be detailed in the following sections.

### 2.3. Histopathology Image Processing

The workflow of WSIs processing mainly comes from the DeepAttnMISL proposed by Yao. J [18]. Due to the gigapixel of the WSIs, it is difficult to calculate them directly. The first step is cutting WSIs into patches with a small size of 1024 × 1024 (pixel × pixel) for calculating in the next step. Based on experience, we chose 20× magnified images for patch sampling. The 20× magnification achieves a balance between cell morphology and overall cell distribution, taking into account both micro and macro information. The patches contain all tissue areas except the background. In the cutting process, there is overlapping between patches, and the patch sampling position is random within a limited range. The number of patches cut from each WSI varies from 100 to 4000. A patient may have several WSIs, and the patches cut from these WSIs are put together. To keep the balance of the patch dataset, we performed data augmentation on the patients with a small number of patches, including flip horizontal, flip vertical, and rotation. For example, if a patient has less than 200 patches, we will perform flip horizontal, flip vertical, and rotation of 90 degrees, 180 degrees, and 270 degrees on each patch, so that 5 amplified patches produce from 1 patch. There are at least 500 patches per patient after data augmentation. More details are provided in Supplementary Materials Table S1.

Features of each patch were extracted by the pre-trained VGG19 [19] deep learning model. The feature is a vector of dimension 1 × 4096. Then, we adopted K-means clustering to divide these patches into 10 categories based on the features. The cluster number 10 is the optimal parameter selected by controlled experiments. Details are provided in Supplementary Materials Table S2. Multi-instance fully convolutional network adopts a generalized Siamese architecture, which is composed of multiple sub-networks with the same structure and shared weight parameters. The sub-network is constructed with two convolution layers and a pooling layer. Each convolution layer is followed by a ReLU active function. The feature extraction part of the sub-network is designed based on the fully convolutional neural network. Therefore, the size of the input layer is more flexible, which is suitable for the research in this paper. Patch features of each category were fed into the corresponding sub-network, respectively, and the representation of each category was output. Through the attention mechanism, different weights were given to different categories. The representations of 10 categories were aggregated to get a patient-level representation that was relevant to survival. In the previous research, it has been found that clusters of interest and image regions can be localized through the attention mechanism, enabling patient-level representations to have strong representational capabilities. Then, the survival risk score of patients was obtained by reducing representation dimension through several fully connection layers.

### 2.4. Loss Function for Deep Learning Training

The model was built based on deep learning that the loss function is the key in model training. The follow-up record of patients usually contains two pieces of information, observed time and censoring indicator. In the model training, the follow-up record of the ith patient was regarded as a label with the format of $\left(t_{i},\;\delta_{i}\right)$, $t_{i}$ is the observed time and $\delta_{i}$ is the censoring indicator which had two values. $\delta_{i}=0$ indicates death not observed, and vice versa. The loss function was designed according to concordance. Assume that the observed time of the jth patient is longer than that of the ith patient, that is, the jth patient is a low-risk patient and the ith patient is a high-risk patient. At the time point $t_{i}$ that the death event is observed ($\delta_{i}=1$), the predicted value of the jth patient’s risk score is $r_{j}$, that should be lower than $r_{i}$ of the ith patient, otherwise, the model will be penalized. The total number of patients is N. The loss function collected patients with wrong predictions for calculating, and then, model parameters were updated by backpropagation. The loss function is shown in Formula (1).

(1) $$L\left(r_{i}\right)=\overset{N}{\underset{i}{\sum }}\delta_{i}\left(-r_{i}+log\underset{j:t_{j}\geq t_{i}}{\sum }\exp\left(r_{j}\right)\right)$$

### 2.5. Pathology Model Training

In deep learning, training set data is used to train the model, and test set data is used to test the performance of the trained model. Therefore, we divide the patient dataset into a training set and a test set with a ratio of 2:1 based on experience. For training, 5-fold cross-validation is a commonly used method. We split the training set into 5 groups randomly. Each unique group will be taken for validation, and the remaining 4 groups will be taken for training. We fitted the model on the 4 groups and evaluated it on the one group. Therefore, each sample is given the opportunity to be used in validation and used to train the model. We saved the model parameters from each round of training. Then, we selected the optimal model according to the evaluation scores and tested it on the test set. More details are provided in the Supplementary Materials Section 3 (Figure S1 and Table S3). Because the model was constructed based on deep learning, the calculation amount and time consumption are mainly in this step. The trained model will output a risk score of each patient as a representation of the WSIs.

### 2.6. Gene Coexpression Analysis

WGCNA is a method to analyze the gene expression patterns of multiple samples that can cluster genes into co-expression modules [20]. It has been commonly used in bioinformatics. In this paper, we adopted WGCNA to get co-expression modules of HCC patients and eigengene, which is the first principal component of each module. The details of each co-expression module are listed in Table S6. In this way, the expression patterns of different gene modules can be highly summarized, and the relationship between different modules and case survival will be analyzed in the next step.

For the collected mRNA-seq data in our study, firstly, the missing values were removed, and then the top 5000 genes with the most obvious variance were picked out based on the median absolute deviation for downstream analysis. Calculate the interaction coefficient between genes, get the adjacency matrix, and then calculate the topological overlap measure. After that, we get 8 gene co-expression modules by hierarchical clustering and dynamic tree cut. The eigengenes of each module were extracted as the representation of the module. To avoid the final integrated model overfitting, we performed the least absolute shrinkage and selection operator (LASSO) to select an informative subset of factors. The description of this process is provided in Supplementary Materials Section 4 (Figures S2 and S3, Tables S3 and S4). 5 modules were retained, and the top hub gene of each module was extracted as a risk factor in the next step. The 5 hub genes are *DCAF13*, *ELAC2*, *ZNF320*, *KIF18B*, and *FERMT3*.

### 2.7. Integrative Multi-Modality Prognosis Model

With the deep learning risk score from histopathology images, module hub genes from genetic data, and clinical characteristics (age and sex), we built a multi-modality integrative prognosis model based on Cox proportional hazards model. The integrated model can estimate survival risk and calculate a comprehensive risk score by which patients can be stratified into a low-risk or high-risk group.

### 2.8. Statistical Analysis and Model Assessment

C-index estimates the probability that the predicted results are consistent with the observed results. The value of C-index ranges from 0 to 1, the larger the value is, the better the model predicts. We used C-index with 95% confidence interval (CI) was used to assess the prognosis model performance. Time-dependent receiver operating characteristic (ROC) curves of 1-year survival and 3-year survival were plotted, and the areas under the curves (AUCs) were calculated. The value of AUC ranges from 0 to 1, the larger the value is, the better the model performance.

Kaplan-Meier (KM) survival curves of high-risk and low-risk groups were plotted, and the log-rank test was used to test the difference between the two groups (*p*-value < 0.05 is considered statistically significant).

The predictive factors, including risk scores from histopathology images, top hub genes of each co-expression module, and clinical characteristics (sex and age) are used to plot a prognosis nomogram. The impact of factors on prognosis can be seen intuitively in the nomogram [21]. Nomogram calibration curve was used to test its prediction performance. Then, DCA [22], which is a method for calculating model clinical net benefit, was used to assess the clinical value of the multi-modality model.

As a controlled experiment, we built Cox proportional hazards model based on single-modality factors and multi-modality factors, respectively. The above evaluations of these models are calculated to prove the prediction effectiveness of the multi-modality model.

In this study, histopathology image processing was performed in Python (3.8.8) and the deep learning model was constructed based on Pytorch (1.10.2). WGCNA and statistical analysis were performed in R (4.2.1). The R package includes WGCNA, dynamicTreeCut, glmnet, survival, rms, ggDCA, survcomp. Details of data and code are provided at github (https://github.com/Houjiaxin123/Integrative-Histology-Genomic-HCC-Prognosis-Analysis (accessed on 24 September 2022)).

## 3. Results

### 3.1. Patient Characteristics

A total of 346 patients (232 males and 114 females) with HCC were included in this study. The median age of patients at first diagnosis was 61.0 (range from 16.0 to 85.0). There were 123 patients died at the last follow-up, and the other 223 patients’ follow-up records were censored. The median survival time was 19.56 months. TNM stages of patients are shown in Table 1. In our study, we used 5-cross validation to test the performance of the integrated model. The dataset was divided into a training set and a test set with a ratio of 2:1. The training set was used to train the deep learning model. The test set is independent. Details of the two sets are shown in Table 1.

### 3.2. Multi-Modality Model Perfomance Evaluation

The C-indexes with 95% confidence intervals of single-modality and multi-modality models are listed in Table 2. To validate the model generalization performance, we calculated C-index and AUC on the training set and test set, respectively. We collected the predictions of the selected optimal model in the training process to calculate the training set C-indexes. The test set C-indexes are based on the trained model predictions of test set samples. Results of C-index difference test between single-modality and multi-modality models are shown in Table 3. The results prove that the C-index of multi-modality model is higher than that of single-modality models with statistically significant. Therefore, multi-modality model has stronger prognosis power.

**Figure 3:**
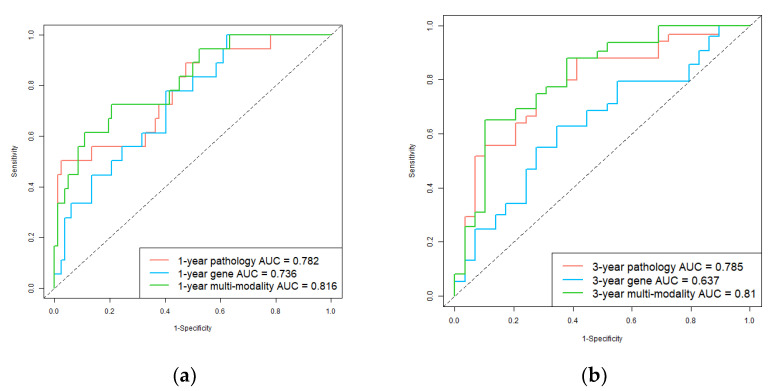
Receiver operating characteristic (ROC) curves of single-modality and multi-modality models in the test set. (**a**) 1-year hepatocellular carcinoma (HCC) overall survival prediction ROC of the models; (**b**) 3-year HCC overall survival prediction ROC of the models. Red, blue, and green plots represent the pathology prediction model, gene prediction model, and multi-modality prediction model, respectively.

In addition, we used ROC and AUC which are more comprehensive assessments to describe the model performance on the test set. The ROCs are shown in Figure 3. For 1-year survival prediction, AUCs of pathology, gene, and multi-modality models are 0.782, 0.736, and 0.816, respectively. For 3-year survival prediction, AUCs of pathology, gene, and multi-modality models are 0.785, 0.637, and 0.810, respectively. The results also proved multi-modality model is superior to single-modality models.

### 3.3. Survival Prediction by Multi-Modality Model

**Figure 4:**
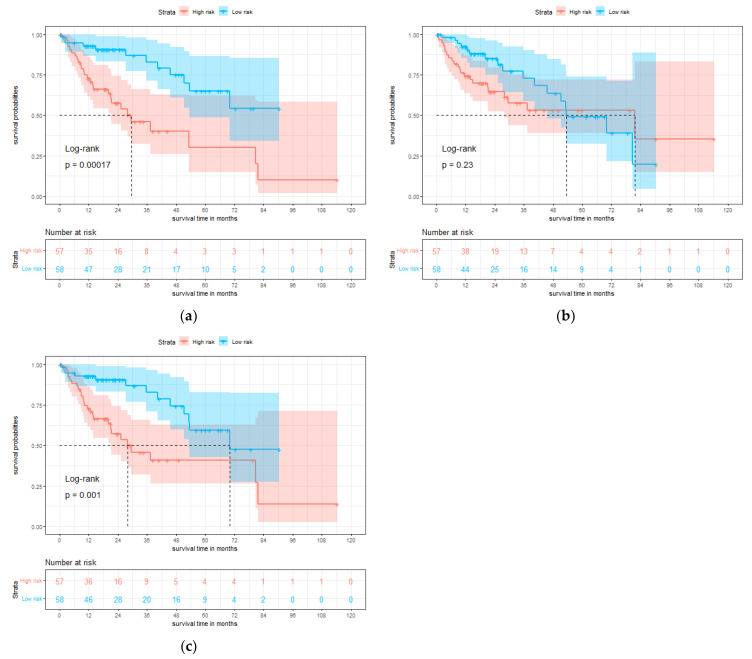
Single-modality and multi-modality models were tested on the test set for hepatocellular carcinoma (HCC). (**a**) Kaplan-Meier (KM) curves of overall survival with the prediction of pathology prognosis model (*p* = 0.00017, two-sided log-rank test). (**b**) KM curves of overall survival with the prediction of gene prognosis model (*p* = 0.23, two-sided log-rank test). (**c**) KM curves of overall survival with the prediction of multi-modality prognosis model (*p* = 0.001, two-sided log-rank test). Red and blue plots represent high-risk and low-risk groups, respectively.

To further assess the model in the test set, we divided the patients into high-risk and low-risk groups according to the median value of the predicted risk score by each model. KM curves of overall survival are shown in Figure 4. The survival rate of the low-risk group is significantly better than the high-risk group, and the result of multi-modality model (*p* = 0.001) is more significant than gene model (*p* = 0.23).

### 3.4. Nomogram and Clinical Benefit Evaluation

**Figure 5:**
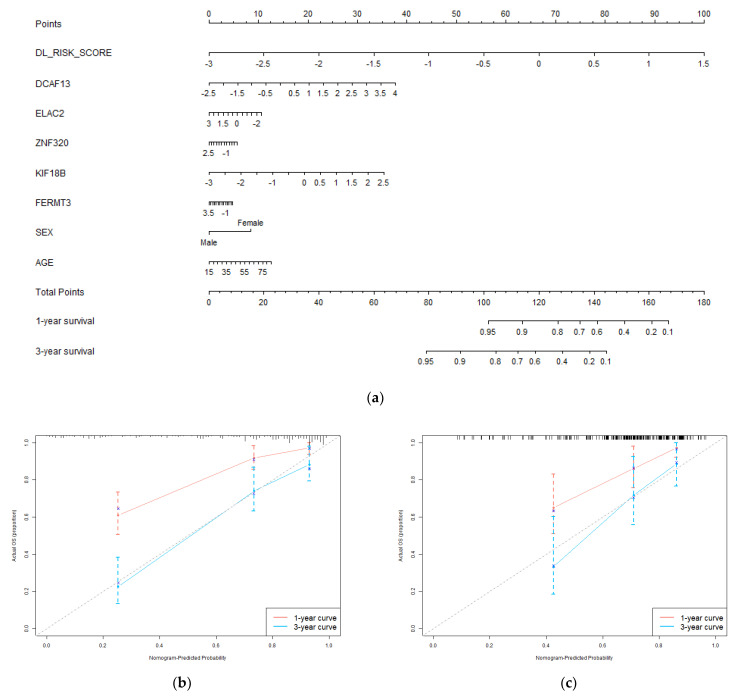
Evaluation of the predictive performance of the integrated prognostic model. (**a**) The nomogram incorporates deep learning pathology risk score, genomic module hub genes, and clinical characteristics of the patients in the training set; (**b**) The calibration curve of the nomogram for 1−year and 3−year overall survival prediction in the training set; (**c**) the calibration curve of the nomogram for 1−year and 3−year overall survival prediction in the test set.

We constructed a nomogram system with deep learning pathology risk scores, hub genes, and clinical characteristics (sex and age) by Cox regression. It integrates histopathology and genomics for 1-year and 3-year survival predictions of patients. The nomogram and its calibration curves are shown in Figure 5. In Figure 5a, different values of each factor correspond to different points on the first axis. According to the nomogram, patients can be scored based on their own factors. The obtained total points can be mapped to the 1-year and 3-year survival probability axis to get prediction results. Nomogram visualizes the prognosis model in the form of a scale, which may be helpful for clinical practice. The calibration curve is plotted according to the nomogram predictions and the actual outcomes of patients. The closer the calibration curve is to the diagonal, the stronger the predictive power that the model has. The calibration curve in Figure 5b demonstrated the survival predictions of the nomogram are close to the actual survival, especially the 3-year predictions. We also processed external validation on the test set, and the calibration is shown in Figure 5c. It proved the integrated model has a good prognosis ability. Nomogram with only clinical characteristics is provided in Supplementary Materials Figure S4. Table S5 demonstrated the prognosis power of clinical characteristic.

**Figure 6:**
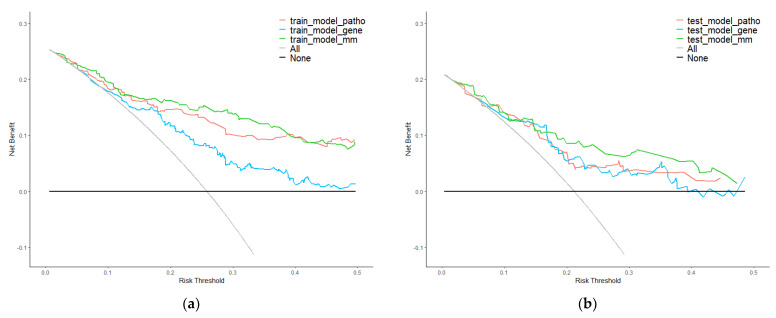
Decision curve analysis (DCA) of single−modality and multi−modality models. (**a**) DCA for models of survival prediction on the training set. (**b**) DCA for models of survival prediction on the test set. Red, blue, and green plots represent pathology, gene, and multi−modality modes. Grey and black plots are two net benefit reference lines in an extreme situation.

Moreover, we used DCA to evaluate the clinical value of the integrated model. The results in the training set were regarded as internal validation and the results in the test set were regarded as external validation. Curves are shown in Figure 6. Whether in the training or test set, the net benefit of multi-modality model was higher than single-modality models.

## 4. Discussion

In this study, we designed a workflow for HCC prognosis that integrated histopathology and genomics to make a more accurate prediction of patient overall survival. For WSIs, we calculated a risk score of each patient by using DeepAttnMISL, which is a state-of-the-art deep learning algorithm. For mRNA expression, we clustered genes into 8 modules by WGCNA and extracted the top hub genes of modules as risk factors for each patient. Then, histopathology risk score, hub genes, and clinical characteristics were integrated by constructing a Cox proportional hazard model. We verified the prediction performance of the integrated model by several evaluations, such as C-index, ROC, and AUC. KM curves were used to show the stratification ability of the model. Whereafter, we constructed a nomogram based on the multi-modality factors and validated the clinical value of the integrated model by DCA.

Aiming at the heterogeneity of cancer, the application of multi-modality data analysis is the trend in cancer study of the occurrence and development, as well as clinical diagnosis and treatment. The experimental results in this paper prove a key point that compared with the single-modality, the multi-modality model can predict the survival risk more accurately. It is very promising to carry out the next research in the direction of multi-modality data analysis.

Pathological sections have always played a necessary role in clinical diagnosis. With the development of digital technology and computer vision, WSIs have gradually become an important modality in computer-aided survival prediction. Genomics contains molecular information, while WSI contains morphology information of cells. Some studies [23,24,25,26] have tried to extract morphological biomarkers from WSIs, which may become an indicator for clinical application. These research also confirmed the effectiveness of pathological images for survival prediction. Through the performance comparison between histopathology modality and gene modality, our study also confirmed that pathological images have prognosis power in the clinic.

Deep learning has gradually become a popular method of big data analysis. Especially in medical image processing, deep learning is a utility for high-dimensional feature extraction [27,28,29,30]. The convolutional neural network has been used for WSIs feature extracting in multi-modality prognosis studies as early as 2018 [31]. Compared with the manually extracted features used in traditional machine learning, deep learning features are more objective and considered to retain more information. However, its defect is poor interpretability, which the difficulties to combine high-dimensional features with clinical phenotypes. In some multi-modality medical research [32,33,34], the extracted medical image’s high-dimensional features are fused with other modalities by vector calculation, which is a relatively effective method used in multimodal fusion at present. Although most studies used visualization methods to combine high-dimensional vectors with visual presentation, the limitation of poor interpretability is not solved fundamentally. In our study, the two modalities are combined by integrating, which avoids some problems of poor interpretability of vector calculation. It is obvious that the risk score calculated by deep learning has a strong prognosis power through the comparison between single-modality models. We can speculate that the network caught the key information in patches by forming a complex nonlinear model of survival risk. However, the integration method doesn’t achieve the deep fusion of multimodal that may need some prior condition settings based on the biological connection of modalities. Multimodal fusion still needs more research to enhance the interpretability of the algorithms.

**Figure 7:**
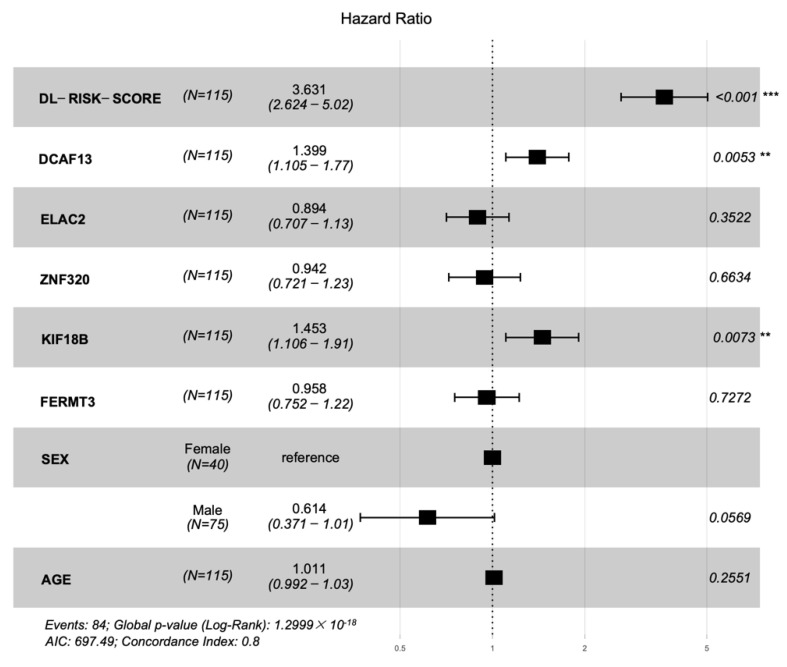
Forest plot of the hazard ratios with 95% confidence intervals (CIs) and *p*-values obtained from the multi−modality model including the deep learning risk score, hub genes, and clinical characteristics of the cohort. The ‘***’ indicated *p*-value < 0.001, and the ‘**’ indicated *p*-value < 0.01.

We plotted hazard ratios (HRs) of factors in Figure 7. The deep learning risk score (HR = 3.631, *p*-value < 0.001), *DCAF13* (HR = 1.399, *p*-value = 0.0053), and *KIF18B* (HR = 1.453, *p*-value = 0.0073) are the factors that most significantly associated with prognosis in the test cohort. *KIF18B* is a member of superfamily proteins [35]. In previous studies, *KIF18B* has been identified as one of the core genes in HCC microenvironment. Upregulation of *KIF18B* was related to poor prognosis [36,37]. *DCAF13* is a frequently amplified gene in various cancers. Cao J et al. [38] considered *DCAF13* plays a potential role in cell cycle regulation. They found and proved overexpression of *DCAF13* is related to poor prognosis. Luo Y et al. [39] also thought *DCAF13* is one of the genes associated with the prognosis of HCC. These findings are consistent with the results obtained in our paper.

Our study also has some limitations. Firstly, the integrated multi-modality model lacks multi-central data to verify its performance. Secondly, the mechanism between gene and phenotype also lacks analysis. In particular, the modeling of the potential relationship between modalities is not clear, and the research results are more at the application level but have not yet gone deep into the mechanism level. Wu. J [40] et al. identified new subtypes across three malignancies by two imaging modalities and proved this discovery by the different phenotypes after therapies. This study is an exploration of the connection between tumor morphology and molecular. In the next step, on the one hand, we will explore more effective algorithms for extracting multimodal data information; on the other hand, we will try to model the connection between modalities and explain the computational representation based on biological discoveries.

## 5. Conclusions

In conclusion, we provided a workflow of multi-modality analysis for HCC prognosis, mainly including histopathology and genomic data. Through the various assessments of the integrated model, it can be concluded that multi-modality information has great potential for cancer prognosis, and the multi-modality model constructed by computational modeling may become an effective tool to assist clinical practice. However, more research is needed to explore multi-modality information extraction and feature fusion methods. The implicit connection between modalities is also a meaningful research topic in the future.

## Figures and Tables

**Table 1:** Patient Characteristics.

Characteristics	Total (N = 346)	Train (N = 231)	Test (N = 115)	*p*-Value
Age: median (range)	61.0 (16–85)	61.0 (17–85)	61.0 (16–82)	0.679
Gender				0.608
Male	232 (67.1%)	157 (68.0%)	75 (65.2%)	
Female	114 (32.9%)	74 (32.0%)	40 (34.8%)	
T classification				0.360
T0-T1	169 (48.8%)	114 (49.3%)	55 (47.8%)	
T2	88 (25.4%)	62 (26.8%)	26 (22.6%)	
T3-T4	88 (25.4%)	55 (23.8%)	33 (28.7%)	
NA	1 (0.3%)	0	1 (0.9%)	
N classification				0.703
N0	241 (69.7%)	165 (71.4%)	76 (66.1%)	
N1	3 (0.9%)	2 (0.9%)	1 (0.9%)	
NX	101 (29.2%)	63 (27.2%)	38 (33.0%)	
NA	1 (0.3%)	1 (0.4%)	0	
M classification				0.177
M0	252 (72.8%)	173 (74.9%)	79 (68.7%)	
M1	3 (0.9%)	3 (1.3%)	0	
MX	91 (26.3%)	55 (23.8%)	36 (31.3%)	
TNM stage				0.487
I–II	242 (69.9%)	166 (71.9%)	76 (66.1%)	
III–IV	85 (24.6%)	54 (23.4%)	31 (27.0%)	
NA	19 (5.5%)	11 (4.8%)	8 (6.9%)	
OS (months): median	19.56	19.56	19.53	0.903
Event				0.654
Alive	223 (64.5%)	147 (63.6%)	76 (66.1%)	
Dead	123 (35.5%)	84 (36.4%)	39 (33.9%)	

**Table 2:** Concordance index (C-index) of single-modality and multi-modality models.

Modality	C-Index ^1^ (Training Set *n* = 231)	C-Index (Test Set *n* = 115)
Pathology	0.776 (±0.054)	0.714 (±0.088)
Hub gene	0.692 (±0.061)	0.666 (±0.081)
Multi-modality	0.796 (±0.055)	0.746 (±0.077)

^1^ 95% confidence intervals (CIs) are in parentheses.

**Table 3:** C-index significant difference test between single-modality and multi-modality models.

Model	*p*-Value (Training Set)	*p*-Value (Test Set)
Pathology to Multi-modality	2.582 × 10^−2^	1.946 × 10^−2^
Hub gene to Multi-modality	4.295 × 10^−5^	1.269 × 10^−2^

## Data Availability

The data analyzed in the study is from public datasets online, including TCGA (http://portal.gdc.cancer.gov/projects/TCGA-LIHC (accessed on 13 June 2022)) and cBioportal (https://www.cbioportal.org/ (accessed on 13 June 2022)).

## Supplementary Materials

The following supporting information can be downloaded at: https://www.mdpi.com/article/10.3390/genes13101770/s1, Figure S1. C-index of each epoch in the training process; Figure S2. The process of coefficients changing with Lambda; Figure S3. Selection of the optimal Lambda; Figure S4. Nomogram of clinical characteristics; Table S1. Details of data augmentation; Table S2. C-index with different cluster number; Table S3. C-index of 5 folds on validation set; Table S4. LASSO sparse matrix; Table S5. C-index of clinical characteristics nomogram; Table S6. The 8 co-expressed gene modules generated by WGCNA.

## Abbreviations

AUC	Area under the curve
C-index	Concordance index
CI	Confidence interval
DCA	Decision curve analysis
HCC	Hepatocellular carcinoma
HR	Hazard ratio
KM curve	Kaplan Meier curve
LASSO	The least absolute shrinkage and selection operator
LIHC	Liver hepatocellular carcinoma collection
MI-FCN	Multi-instance fully connection network
ROC	Receiver operating characteristic
TCGA	The Cancer Genome Atlas
WSI	Whole slide imaging
WGCNA	Weighted gene co-expression network analysis
