# Supplementary material for: Integrative Histology-Genomic Analysis Predicts Hepatocellular Carcinoma Prognosis Using Deep Learning
Source: Genes (Basel). 2022 Sep 30;13(10):1770. doi: 10.3390/genes13101770 (PMC9601633; doi:10.3390/genes13101770)
Supplement: Supplementary file 1 [file genes-13-01770-s001.zip › genes-1888547-supplementary.pdf]

genes

MDPI

Supplementary Materials

Integrative Histology-Genomic Analysis Predicts Hepatocellular Carcinoma Prognosis using Deep Learning

Jiaxin Hou, Xiaoqi Jia, Yaoqin Xie and Wenjian Qin

Methods

1. Data Augmentation of Patches

We cut patches from whole slide images (WSIs) of all tissues (benign and malignant regions). The patch number of a patient is determined by the tissue size in the pathology section, the size of WSI, and the number of sections. As a result, the patch number of a patient ranges from 102 to 3655. To reduce the impact of data imbalance on the experiment, we performed data augmentation of patients with a small number of patches. We divided 4 intervals according to the patch number, and different intervals adopted different data augmentation operations, including flip horizontal, flip vertical, and rotation 90°, 180°, and 270°. Details are shown in Table S1. In this way, the number of patches increased by 6 times, 4 times, 3 times, and 2 times. The patch number ranges from 505 to 3655 after data augmentation. Based on experience, it is appropriate for a patient to have at least 500 patches for the experiment.

Table S1. Details of data augmentation.

Patch number (N)	Augmented patch number (N)	Methods
N < 200	600 ≤ N < 1200	Horizon, Vertical, Rotate (90°, 180°, 270°)
200≤N < 300	800 ≤ N < 1200	Horizon, Vertical, Rotate (180°)
300≤N < 400	900 ≤ N < 1200	Horizon, Vertical
400≤N < 500	800 ≤ N < 1000	Horizon

2. Cluster Number Selection

For K-means clustering of patches, the number of clusters need to be set up in advance. To find the most appropriate number of clusters, we performed experiments with cluster numbers of 6, 8, 10 and 12. All other settings and architectures are kept the same. In Table S2, we list the validation C-index in the training process (details of calculation are provided in Section 4 in Supplementary Materials). When the number of clusters is 10, the average value of c-index is the highest. Therefore, we clustered the patches into 10 categories in the manuscript.

Table S2. C-index with different cluster number.

	c = 6	c = 8	c = 10	c = 12
**Fold 1**	0.704	0.707	0.728	0.708
**Fold 2**	0.675	0.643	0.637	0.637
**Fold 3**	0.567	0.556	0.646	0.613
**Fold 4**	0.644	0.677	0.660	0.672
**Fold 5**	0.685	0.681	0.695	0.694
**Average**	0.655	0.653	0.673	0.668

3. 5-Fold cross validation to select the optimal model

In the training process, we performed a 5-fold cross-validation. We split the training set into 5 groups randomly. Each unique group will be taken for validation, and the remaining 4 groups will be taken for training. We fitted the model to the 4 groups and evaluated it in one group. We saved the model parameters from each round of training. Then, we selected the optimal model according to the evaluation scores. Figure S4 plotted the C-index of each epoch. The fold 1 model has the highest value of C-index in validation, and C-index keeps rising and gradually becomes stable in the training process. Therefore, we choose the fold 1 model as the best performance model and test it on the test set. The test results are shown in our manuscript.

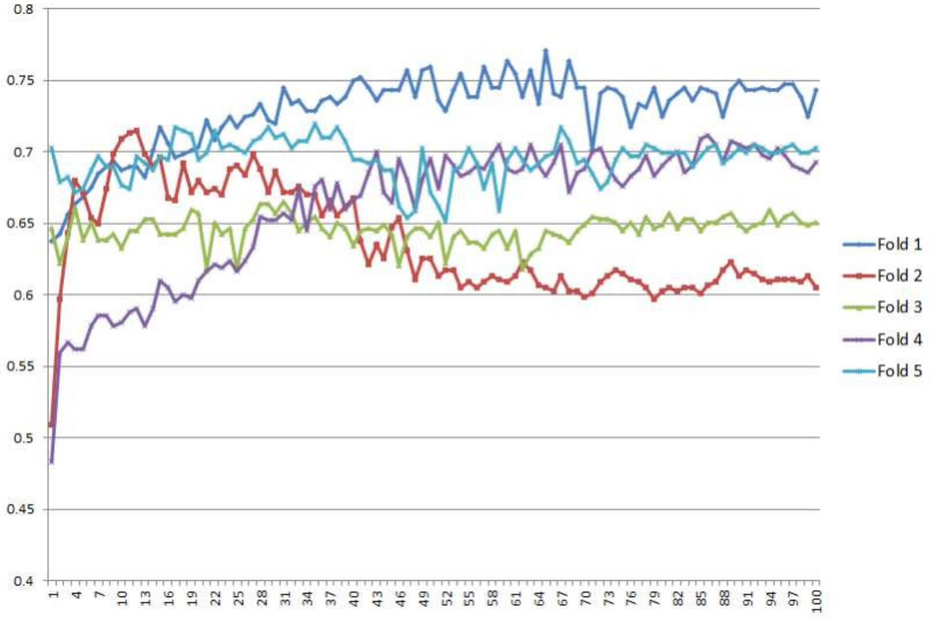

Figure S1. C-index of each epoch in the training process.

Table S3. C-index of 5 folds on validation set.

Fold	C-index (Validation set n=46)
Fold 1	**0.728**
Fold 2	0.637
Fold 3	0.648
Fold 4	0.660
Fold 5	0.695

4. LASSO Regression Variable Screening Process

In our manuscript, we get 8 gene co-expression modules by weighted gene co-expression network analysis (WGCNA). To avoid the final integrated model overfitting and select the modules that are most associated with prognosis, we performed the least absolute shrinkage and selection operator (LASSO). We set the Cox regression model as the target and performed a 10-fold cross-validation. As a result, 5 modules were screened out. The process is shown in Figure S1, Figure S2, and Table S3.

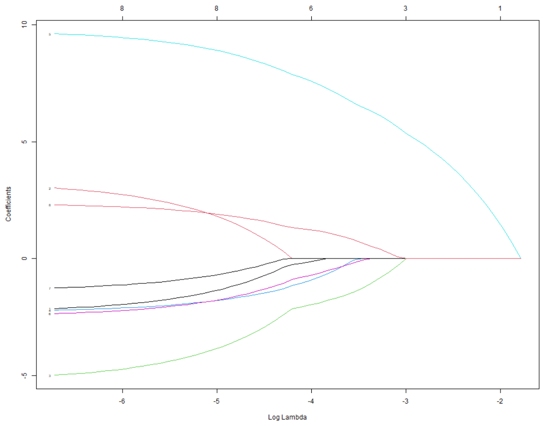

Figure S2. The process of coefficients changing with Lambda.

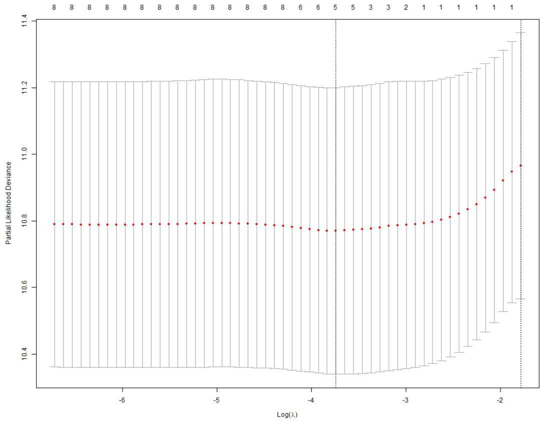

Figure S3. Selection of the optimal Lambda.

Table S4. LASSO sparse matrix.

Module	Coefficient
MEblue	-
MEbrown	-
MEyellow	-1.6891495
MEred	-0.4800721
MEturquoise	7.0979177
MEpink	-0.4152805
MEblack	-
MEgreen	1.0209583

5. Nomogram of Patient Characteristic

To demonstrate the prognosis power of clinical characteristics, we constructed a nomogram with sex and age by Cox regression. The other clinical characteristics, such as TNM classifications, were incomplete for some patients, so they were not included in the model. The nomogram is shown in Figure S5. The prediction C-index is listed in Table S5.

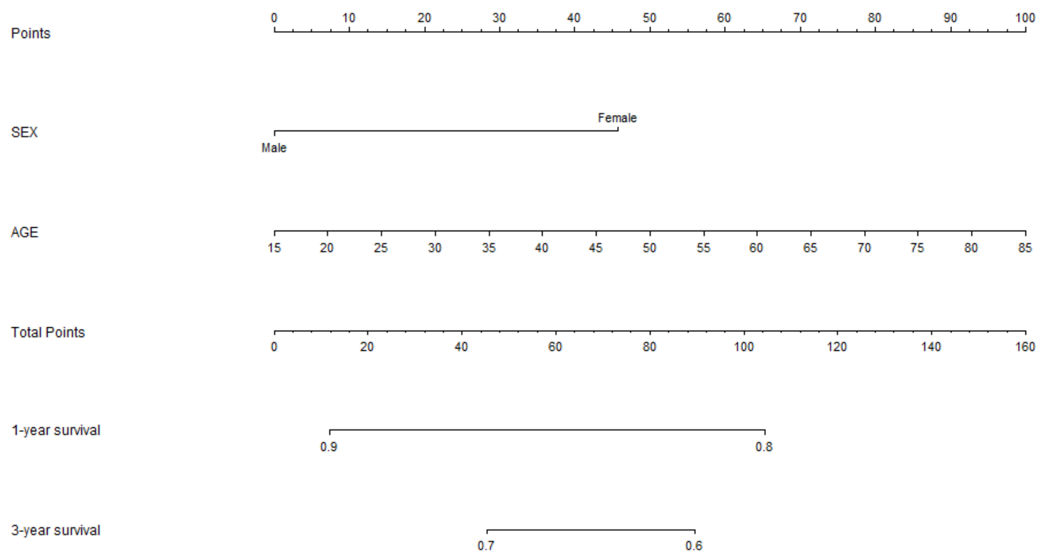

Figure S4. Nomogram of clinical characteristics.

Table S5. C-index of clinical characteristics nomogram.

Modality	C-index[1] (Training set n=231)	C-index (Test set n=115)
Characteristic	0.549(±0.072)	0.504(±0.094)

[1]95% confidence intervals (CI) are in parentheses.

6. Genes in each WGCNA Module

Table S6. The 8 co-expressed gene modules generated by WGCNA.

Module Name	Genes
Module 1 (turquoise)	TMOD4 PTTG2 HIST1H4D ACRV1 HERC2P4 HIST1H2AL C9orf53 HORMAD2 PITX1 ZIC5 MATN1 PLGLA CHRNA4 PEX5L RNF133 FOLH1B GPC5 WDR65 ACOXL RPL34.DT HRNBP3 DMKN SLCO4C1 SEC14L3 CTNND2 DLX4 HIST1H1B FAM155B UGT1A5 LOC399815 BEX2 MIR924HG MSI1 FAM7A3 ZNF488 THRSP IGF2BP1 PEG10 PRR11 SYT1 C1QTNF9 LOC256880 ZNF648 CYP1A2 RAET1K TTC36 MOGAT2 CES7 GPR120 PPAP2C WNK2 ARHGAP11B DEFB132 COL4A5 C5orf27 TRAM1L1 C4orf47 RGS9BP BCAS1 ZNF878 COL25A1 GCGR OTX1 DUSP5P SCUBE1 TREH FXYD2 DLEU2L EFCAB6 C1orf105 SMPDL3B DSCR9 MOGAT1 GREM2 DSG1 LYPD6 CXXC4 ELFN1 RHCE SPATA18 C14orf162 TPPP2 CKMT1B GABARAPL3 LOC100134368 CTSL2 LOC441666 FAM83A.AS1 TMEM232 SNAP25 LOC641367 MAPK13 NLRP6 NCRNA00093 KCNMB2 RDM1 LCTL SALL2 ZNF826 MIP RACGAP1P SGCE NRCAM HIST1H2AH EPCAM LINC00628 FAM72A SULT1B1 TMEM61 MYOM2 RAD54L KBTBD11 HEPACAM BET1P1 C1QL4 CREG2 CHST10 KCNE1L MCM10 PIF1 C15orf42 SLC22A12 CYP3A43 SLC13A3 AVPR2 ADRA1A MTHFD1 FAM57B RAB34 SNHG4 IGF2BP3 GNAT2 ASB4 IQCD RIBC2 PTTG3P CYP2B7P1 SALL4 NHLH1 NRSN2 DRP2 ZNF239 C14orf68 FAM116A STOX1 CLDN2 IAPP INS.IGF2 ZNF257 HIST1H2BO ESYT3 NEB C13orf1 MYO1A HAO2 UBE2QL1 CDCA7 TRIM59 CES8 LINC00634 KIF11 OPN1SW SLC25A25 MYADML2 SPINT1 CELSR3 CYP2A6 KNTC1 SRD5A2 SFN PACSIN1 GINS4 LINGO4 GINS1 HADH C6orf141 C1orf106 ETV4 TCP11 SLC29A4 HIST2H3C ADAM22 ZNF93 CCT6B

	LINC01091 ADRB1 ASTN1 SCP2 CENPQ BCO2 FANCD2 KIF15 MMP1 GPRC5D TMEM145 FANCE GAL3ST1 CANT1 BLM SCARNA12 STAG3 FAM13A RAVER2 APOC1P1 IL1RAPL2 FOLR1 CDKN2BAS KIF18B ORC1L SIX4 DSCR6 CCDC99 LMNB1 VIPR1 DHDH C14orf182 IGDCC4 ESR1 UPP2 ABHD1 GRHPR KIAA1524 ELOVL3 TPRG1L MAGED4B H2AFZ UBXN10 TOP2A EPPK1 CD300LG CCNI2 C8orf39 CYP3A4 FAM64A AFARP1 MCM3 HDC INSIG1 FOXK1 PER1 ACE2 ORC6L GJA9 C17orf53 ECT2 SHOX2 PTBP1 CSPG5 PDZD7 STMN1 FMO1 PACRG FEN1 MYBL2 CYP1A1 FAM54A SLC39A1 CBLN1 SAA2 C6orf138 VPS45 CENPK UGT1A4 GPD1L POLQ CCDC158 TPRG1 POLA1 IL12A AKAP3 SGOL1 NVL TRIM17 C16orf59 KLHDC8A FOXQ1 EME1 MCM2 TK1 ACCN2 ZNF296 FRMD1 NRG3 PIGY ADCY10 SYT8 FAM72B TIGD2 ZBTB7C ALAS1 GLIPR1L2 TBP FGGY RANBP3L MPZL1 PIGV MCM5 RFC4 TTLL4 SCXB ATOH8 CLSPN RTKN2 SFTPD ZMYND12 C2orf48 SLC6A10P MYB RAD51AP1 ZNF18 DBF4 UGT1A3 CYP39A1 SLC22A1 CDC7 FGD1 CRY2 ZNF233 HELLS C16orf70 SUCLG2 EFHD1 SPDYC ZMYM3 GADD45B BRCA1 HIST1H4E SOX4 MAFG FGFBP2 GRIK5 KIF18A FDX1 SLC22A15 MYLK2 FBXL18 CDCA4 SAT2 POLE2 CCNF OR2B6 TRIP13 INPP5J PRIM1 E2F2 NASP HSF2BP IPO9 TK2 TYMS TUBG1 STIL C8orf40 HCG18 TCF3 C21orf34 FANCG PLXNA1 LRP11 LRFN1 CENPW CDC25A CDCA5 INTS7 RAD51 ATAD5 CBR4 CDC42BPG ERCC6L SLC22A20 PDSS2 DUSP1 ACAD11 SPC24 PRIM2 H6PD MPP4 ABCC9 TYRO3 BSDC1 C12orf48 FER1L4 KIAA0226 SENP8 SPHK1 FNDC8 CDKN2D HGFAC RAPGEF3 GTSE1 CENPH GADD45A POLR3C PKMYT1 C17orf28 AZI1 THNSL1 PQLC1 WDR62 BUB1B EML4 SFRS13B COX18 TMEM82 ACADVL SPG7 CDT1 DHRS1 GUSBP11 TACC3 TPX2 MDK KIAA0907 RAD54B FOXM1 MLYCD CENPI MELK C16orf80 C11orf93 MCM4 PEMT MED20 WDR88 GOT2 PLK1 SLC6A8 UXS1 MESP2 GNA14 C17orf69 HMGCL NCAPH RCL1 CDCA3 WHSC1 XPO5 ANKRD24 MAGED4 SEC61A1 ERC2 KIAA0101 CEP250 SPRYD4 C16orf75 MKI67 UBTD2 UHRF1 FAM40B SHCBP1 DLG2 MSRA PSMD14 GMNC NFKBIL2 MLF1IP RCAN3 GPSM2 PRR7 TMEM65 LHX4 CA9 CDC37L1 ZC3H12B MFI2 ZNF207 DENND4B HERPUD1 RAB3D LCAT MYCL1 MYCBPAP RNF24 ZNF883 CDCA8 HIST1H3C ARHGAP11A MFAP3L ST3GAL1 TMC2 CDKN2C E2F7 PTGES2.AS1 NAALADL2 CDCA2 AKTIP DSN1 ZNF530 NAA40 PYCR1 ASF1B IRS2 PLCH1 FANCB C7orf58 PI4KB CASC3 CDC45 FOXJ1 NUP85 TROAP WDR53 TCF19 KIF2C BRP44L CFHR3 HMOX2 KIF7 DEXI RNF8 TIMM17A RNF125 SHB E2F1 SEC14L2 CHAF1B PPA2 GFOD2 CKAP2L PRKCI KCNC1 DEPDC1 DNMT3A C9orf103 UPK3A ABCA8 ZNF300 DEM1 GOT1 RANBP10 DIRAS3 VAT1 NUDT7 C6orf167 NFYC GPIHBP1 LSM11 C9orf172 CDK1 C1orf77 HNRPLL C20orf117 MCM8 PHF19 SIK1 GLOD5 CHAF1A MID1IP1 LIG1 TPT1 CENPE AMD1 LOC401052 NFRKB PAFAH2 POLD1 3-Sep ESD FXN GCDH N4BP2L1 SLC25A30 TXNIP XRCC2 GINS3 NEIL3 GSG2 PSMC3IP CENPO PAK6 UBE2D3 CBFA2T2 TMPO.AS1 SFXN1 RACGAP1 PDE2A REM2 CD14 GAD1 CENPF TMEM175 PRR18 SLC35B2 OBFC2B SMC4 PBX2 SOX12 NUDT6 SOCS2 C11orf1 DLK2 RNFT2 MAPRE1 C8orf46 SCAMP5 TMPPE PTPN23 SPHAR SRL GPR137C DTYMK LINC00205 C12orf32 ABHD14B LPIN2 C10orf32 PCSK1N CYB5D1 TTC13 PGS1 FAN-CI ABCB9 BLVRB TMEM48 TRA2B MSTO2P ISCU FRAS1 GALNTL2 MMAA FBXO43 LAMP1 C15orf21 AURKB CEP55 RBM10 ZKSCAN3 LIN9 SUV39H2 ACYP2 SKA3 CIT MGC12982 E2F8 C14orf80 EIF5 C21orf56 SLC24A5 MED28 MTBP ATXN2L KCNMB3 CPEB3 ARHGAP33 CHEK1 ATAD2 DUSP12 CYP2E1 HAPLN4 KCTD7 H2AFX KIF20B CEP72 GRK6 NUP43 SIK2 ZSWIM1 TLL2 GABARAPL1 DNMT3B PRTFDC1 SLC1A2 FAM83D C6orf208 HDAC11 FAM72D NUDT17 DBF4B FSD1L MAD2L1 FXYD3 ALS2CR4 KIAA1614 DGCR8 BAIAP2L2 MCM6 SNAP47 STRADA ZNF789 ZNF782 YEATS2 CCDC14 ITGB3BP KIAA0564 TERF2IP ALG5 CKS2 NSUN6 PAQR4 LOC642846 AIFM1 TSC22D3 MDH2 ZNF618 GABARAPL2 COQ9 MAST2 CCNB1 EPR1 ACOT1 HRSP12 SCN4A C19orf26 MTA3 PRKCD LAP3 RNF152 FANCA KRBA1 SLC6A13 TFRC PKN3 PLK4 C4orf46 NAT2 FNDC3A GRHL1 FAM24B POLR3F BEND3 NEURL3 FAHD1 TOE1 AATF GINS2 GMPS SETD4 FUT2 TMEM88 KCNJ8 MKKS PPHLN1 PEPD FBF1 QDPR KIF23 PLIN1 SHPK ACACA ZNF692 LASP1 LZTS2 CBR1 SUMO2 FCN2 SRD5A1 GHITM SAE1 ESCO2 CPT2 C19orf36 SMG5 CCDC88A C4orf34 SOD1 CISH

	AURKAPS1 RTDR1 NDUFAF1 KIAA0415 ST6GALNAC6 HEATR6 NDE1 REEP2 ANKRD36 FAM185A MCM7 TTC27 KDM5C SLC20A2 TTK PEX11A EXOC7 GPT2 E2F6 RWDD2B SAA1 TBC1D22B C9orf140 GPRIN1 ASXL1 CYP26B1 PGBD1 NCRNA00107 RNF39 DTL COG3 C20orf112 IGSF1 EDC3 MYO19 HNRNPA3 ZNF519 PASK ECHS1 SPIN4 LOC339047 RHOB PHTF2 DCAF16 MAZ BAT1 PEX11G FLJ37201 RAD51L3 NPAS1 C17orf44 LUC7L3 TUBB PA2G4P4 TRPC1 BIRC5 CBX3 PTGES3 DNM1P35 MKNK1 DDX12 SNRPD1 VPS36 JMJD1C.AS1 DAK PHIP MYO16 CCDC40 IQCC FUS C9orf25 DKC1 SPPL2A SFRS8 CKAP2 GPR19 FLVCR1 C16orf93 ADRA1B PRPF3 C17orf108 BUB1 PVRL1 DNAJC9 DNASE1L3 CYP11A1 STARD5 PARP1 ACOT6 CDC6 ACAT1 AVPI1 CASKIN1 SKA1 HMGN1 SNHG3.RCC1 ADAT2 HSD11B1 DRD1 MYBL1 MT1X ADI1 KIAA1841 TRIM25 UNC119B CDKAL1 PARD6G C1orf135 SSR1 WDR34 ACAA1 ARHGEF2 C7orf44 ALDH2 METTL9 UHRF1BP1 ANAPC7 TMEM192 CBX1 OCA2 LBR RFC2 HNRNPA3P1 NFYC.AS1 R3HDM1 ZSCAN20 ACLY C4orf21 MRPS31 GABPB1.AS1 MGST3 C5orf54 FBXO45 SGOL2 CCDC28A HMGB2 KIF24 NUSAP1 SLC36A1 POLA2 UBE2T FITM1 AMFR ECHDC2 LASS5 RERE TRIM37 C1orf183 ZNF620 TPM3 WDHD1 MAT2A MRPL32 RCE1 DNAJC25 TMEM106C CASQ2 TRAIP ZFP64 HIBCH C9orf100 CCDC150 UBE2Z PTS L3MBTL PDK4 VSIG10 COPS7B UFSP2 SLC1A4 ANKRD32 DCAF11 HMGXB3 NPLOC4 PURB C7orf49 MCM3APAS FOLH1 CHORDC1 NFYA C1orf116 C17orf86 ZMYND10 ODF3B ABCC11 C21orf45 WHSC2 DPY19L2P2 HDAC6 ZNF286B C21orf58 ITPKA BTF3L4 CCDC34 ILF2 DHODH AASS RBM19 TAPT1 TUBD1 HAGH ABCG2 HNRNPU ASAP1 MSH5 DCXR
Module 2 (blue)	CEP170L HRH4 GPR21 IMPG1 TAS2R4 OMP C9orf128 GPR179 GNRHR LDHAL6B NLRP14 NT5C1B C4orf36 CLDN20 CNKSR2 NPIPB13 CSNK1A1L LINC00598 SYS1.DBNDD2 CXADRP3 FAM66D CRHR2 C15orf28 RASEF CDKL5 FLG HCG2P7 C1orf223 KCND3 SNORD116.20 DLEU7 BTC MGC16384 CDH8 GABRR2 GPR20 RPS15AP10 ETV3L FOXP2 SLC10A6 C12orf74 C6orf186 ASAH2B BEND3P3 RPL23AP64 BTBD8 PCDHAC2 PAR5 SMG1P1 RP1L1 NNAT LPAR4 PGM5P2 EBLN2 PCDHGA2 ROR1 PCDHGB3 CLDN16 PAPPA2 LOC402377 TAS2R5 PCDHA6 ESPNP KRTAP5.AS1 ZNF366 EDIL3 GDF7 CACNA2D1 OCLM NFATC2 TSPYL3 TIGD4 UBE2Q2P2 ZNF354C GOLGA9P TNFSF18 BMP8B ZNF625 NTN3 ZNF699 ZDHHC19 KIRREL TSSK4 RUNX1T1 NIPAL1 SNORD116.28 FAM157A PCDHGB5 SNORD116.4 IMPG2 C1orf173 SNURF NBPF16 LINC02995 ERAS TSSK1B ZNF829 C9orf47 LOC100272216 ITGA8 MORF4 GGT8P CYSLTR2 CCDC144A SLC5A4 ARID3C PCDHGA6 TAOK1 CRYBB3 HIST2H2BA CCDC144B ZNF827 EIF3C SLC30A4 OXCT2 POTEE RAB27B CORT PCP2 SLED1 THEM5 PLXNC1 TEKT4 CCDC62 EBF2 GPR17 TTLL7 C1orf161 LOC90834 CALML6 PCDHGA5 ANKRD20A4 TRIM78P SUCNR1 CHRM5 PCDHGA1 P2RY1 AVPR1A PYY2 C1QL3 DNAH11 HYDIN HS3ST3B1 LOC100170939 SLIT2 IQSEC3 PCDHB17 C9orf102 PIWIL2 SNORA39 MAP3K14.AS1 SPATA1 IPMK CDC14C B3GNT5 TAS2R14 PCDHGA9 PCDHGB2 RGS7BP TMED7.TICAM2 PCDHGA10 LCOR CTAGE10P SCARNA5 TYW1B ZNF430 YY2 UBE2Q2P1 P2RX3 CD109 UHMK1 GPR75 RAET1E UBXN7 ADAMTS15 ANKRD36BP1 PCDHGB1 KIAA0754 ANKRD36B BARD1 SPATA6 LRRTM2 PRKG1 SERTAD4 LOC728989 MICALCL RXFP1 GOLGA6B IKZF3 LOC100190986 EHF CALR3 UNC5C TEX9 GPR89C CLOCK SSR2 C4orf12 OR2A4 CAPS2 NPR3 PCDHGA12 CCDC39 TXNDC6 CHRM3 ANKRD44 RASA2 ZDHHC20 HDX PCDHGA11 USP51 BTBD19 MACF1 INHBA IGSF9B NHSL2 C14orf174 USP45 MPZL3 PFDN2 AK7 ATMIN PDE3A KLF7 SYNC STAG3L1 TRHDE ARHGAP20 REL LRRC37A6P MCTP1 KLHL11 H2AFB1 SEC14L1P1 HSN2 C6orf155 RABEPK PCDHGA3 GAS2L3 MYO9A MAP3K2 C1orf95 MAK FAM63B TNNC2 MRPL9 BCLAF1 C20orf152 ZNF609 CASS4 DLEC1 MGAT5 ITGA2 SLC2A14 WFDC3 ADAT1 MAN1A2 HAX1 GATSL1 RGPD5 ITGB3 ZNF37A C2CD4D.AS1 MORN2 HSD17B3 PTAR1 GOPC ANKRD20A9P PCDHGA7 WIBG C12orf10 NCKAP5 ALS2CR8 C10orf110 ZNF32 RC3H2 PCDHGB4 PPP2R4 PARD3B TXN ATP10D B9D1 FAM13C C6orf204 BHLHA15 TMEM85 GBE1 NCRNA00171 SON PCDHB11 POMK PAK2 UBQLN4 DOPEY2 PCOTH LOC728554 PCDHB13 ZNF511 TMEM183A ERLIN2 CSRNP3 ZC3HAV1L TNNI3K MTMR9 C20orf94 ZNF284 CDKL1 ZNF843 PLAC8L1

ADAR PCDHGB6 MRPS21 THAP8 ZNF770 CAPN12 LNX2 HSPC157 NFAT5 MBTPS1 FAM54B
CITED2 PCDHGB8P DOCK5 MIER3 ZKSCAN1 PCDHB8 BAZ2B DTWD2 EEA1 TNRC6A
TECTA KCNQ4 MXD3 MYSM1 KLHL5 SNRPA1 ST3GAL2 PRCC CDK5 C15orf33 SLK PNN
RBM25 ACBD6 C9orf89 QSER1 NSDHL C6orf81 HIST4H4 DMXL1 ENTPD7 MDM4 SPDYE1
LMBRD2 ARPC3 DNAJC6 SEC61B FAM73A LOC728743 RAG1AP1 ZNF619 SERINC3 MFN2
PDRG1 UTP14C DDI2 TTBK2 KLHDC1 MRPL34 C1orf182 DOPEY1 CMTM6 AIFM2 GOLGB1
LONP2 USP32 CCNT1 NOP16 GPX1 NACA ARL10 BCL8 STX4 BRWD3 CLIP1 ATP6V1B2
WDR31 ZMYM2 PCM1 MICAL3 SGMS1 PHKB HUWE1 WRN MBTD1 COASY CCDC17
CLIC4 SPATA24 ANO3 TOR1AIP2 RAB33B KIF5B DGCR6 FLCN ANAPC1 C17orf59 RPGR
GDPD1 HRNR RCOR3 RFX7 NBEAL1 NCRNA00204B ACER2 TTLL9 POU2F1 TNPO1
TBC1D17 RSPH3 SLC38A2 TBC1D2B CREBBP PCDHGA4 SNRPF GBAP1 XRN1 PDPR2P
SFRS18 TELO2 MAP3K13 MCTS2P EIF2B4 UBA6 C1orf220 PIK3R1 THAP6 C21orf33 ZNF483
SPTBN1 BAG5 DDIT3 PGP VPRBP RNF113A STRN IKBKB POLR3H ZFYVE19 ZNF426
SMAD9 GORASP1 CD99 OTUD7A MPDZ ATRX CLTA TRIM9 NMD3 ADCK1 GNA13 CNPY2
N4BP2 RASSF6 C3orf37 ARNTL OVGP1 IDI2 KIF1B UQCRH DNAJB14 WASF3 ANP32A
PHF20 STOML1 GALK1 CDYL DCAF12 11-Sep NUTF2 SH2B3 ZBTB6 ZNF704 ARHGAP42
SAMD8 FBXO21 SLC35E2B ZNF516 SNRPG ZNF37B ANP32B CCDC58 POGZ UQCRHL
C10orf118 C5orf41 RPL21 AFF1 PSMB4 PFDN4 RAD54L2 MAGOHB KRTCAP2 RBM34 LUZP1
ZNF774 SESN3 MRPL18 KLF3 APOOL NRL NR1D2 TMEM151B TRIM44 C2orf68 CAPN7 PPIB
ERN1 TULP4 AP1G1 FAM76B RAPGEF5 DNMBP ATP5J HIST1H1C INTS2 C17orf57 HEXIM2
DYNLRB1 SMG1P3 ANKRD39 PCDHGB7 HDGFL2 TTC21B DENND1B DENND4C CDK10
PSMA4 NSMCE4A SNRNP25 IGF2R MRPL36 PSMD4 RIOK3 DLG5.AS1 MYLK KIF22 NLGN4X
ABCB8 CNTF SOCS7 ARHGEF12 FBXL3 NHLRC2 CHD6 CP110 GATAD2B GPBP1L1 BMS1P4
SLC4A7 SLC5A3 ZEB1 PDIK1L ZFP36L1 FOXRED1 SSBP1 ANKHD1 ASNA1 MRPL1 C20orf24
GIPC1 SATB1 THRAP3 HMBS BMP2K ZYG11B HTRA2 ARL5B GLRX2 NDUFA1 NR6A1
C4orf41 KLHL15 BCRP3 THAP7 GOLGA1 C3orf67 KLF11 CCDC153 BCAP31 GAPVD1 NXT1
USP46 TIA1 CNOT7 FAM122A ZNF680 CEP120 NFKBIZ BIRC6 FAM179B FAM83C NAPEPLD
C2orf28 DHRS13 MKL2 ZNF81 AR TMEM91 C1orf31 MAML2 LAGE3 EDEM1 WAPAL
BNIP3L SRBD1 ATE1 ZNF646 SENP1 USP49 STARD4 PEX16 RANGAP1 DDR2 FGF2 THAP1
MYST4 PPFIA2 UFD1L CTU1 PHACTR2 COL4A3BP ERP44 BRI3 GCNT4 DBT PPIAL4G PPIL4
TUBGCP4 THOC7 CENPP ZMIZ1 MBLAC1 SLC2A13 P2RX7 C20orf177 DCP1A ITGA6
TMEM181 ABL2 WDR25 PPM1L KBTBD3 NUCB1 VPS13D LOC100128842 PDS5B UFC1 CRTC2
DRAP1 CLCN3 RPL22L1 DPCD GAPDH CYLD LARP4 SNORD1C HSPE1 UBR4 SAP130
ZNF280C EIF2C3 MECR RMND1 OGT ZBTB17 ATF4 C1orf192 SCAPER NACAP1 EXOC6B
ZDHHC2 C2orf64 WBP1 EME2 GFM1 DNAJB2 APPBP2 ZBTB38 SERTAD2 FAM59A DPP8
PLEKHA1 SF3B6 MAGI1 MAGOH BAGE2 TYSND1 DAAM1 TRPT1 MRPS11 PQLC2 BMPR1A
CCDC163P DCUN1D3 CYTSA KIAA1530 SFMBT2 TMTC1 VPS54 NRP1 NORAD CKLF
TBC1D3C ABTB1 ATPIF1 MLL2 KDM6A MED23 HEATR5A DAG1 HECTD2 LSM10
DKFZp686O24166 ZC3H18 ATF7IP GEMIN6 LOC388955 CNPY3 IDE SCART1 NUMB SHPRH
SMG1 STOX2 ZNF804A BAZ1B MAP4 ORMDL2 WNK3 POMZP3 ITPR1 DIDO1 LYSMD4
CELSR2 C1orf122 MAN2A1 PREX2 PIGN MCPH1 ZBTB48 SLC12A6 PROX1 BRE PPP2R2A
GK5 SPR DPH3B NAB1 NDUFB8 TIRAP GPRIN3 FBXW8 COQ4 C19orf71 CTSA FKBP14
C13orf31 RER1 NUPR1 TOMM5 LINC01881 BICD1 NME2P1 PCGF5 ZFYVE20 DOHH
TOMM40L TMEM30A ITGB1BP1 C22orf27 NUBP2 PHF16 UACA CEP350 DENND4A EIF2S2
PDCD5 ZNF436 HSPB11 ZNF616 C14orf4 POLD2 CCDC59 VPS25 ROCK1 KLRA1 DNAH10
PPP4C CHD9 PI4K2B CALR IRF3 NOTCH2NL PCSK5 BAT2L2 WDFY1 C9orf169 CCDC142
CETN2 HINT2 LOC729603 NDUFB4 C16orf52 FAM128A IL1RAP PDXDC1 PPP2CB ATP5G1
FRS2 INO80E MBD3 SERINC1 ZMPSTE24 ZXDB POLR2H SLC25A39 CMIP GTF2A2 EPB41
ZBTB26 ZNF445 PSMA3 UBXN2B TMEM53 HINT3 DCLRE1A B3GNT2 ECE2 CDON TLCD1
PAPD5 TMTC3 LOC401093 NDUFA12 LRRFIP1 ZAK C14orf166 PSME1 AKAP11 ASH1L
PCDHGC5 BAG4 SIGIRR SMURF1 BIRC2 CHKB.DT BOLA3 PDF BRIP1 SEC14L4 FRK UBC

	C15orf40 TMEM60 MRPS33 PMF1 DDHD2 CSDE1 ZFP91 C18orf22 LENG8 PHF3 C14orf181 COPZ1 NDUFA9 ALS2 TPRKB GCC2 AGAP3 DNAJB11 RASGEF1B DGCR14 ZNF33A EIF6 SYNJ2BP GTF2H5 LINC00893 FAM100B TNFAIP1 DLG5 NDUFA6 ZMYND19 PPM1A PTPN12 SLC33A1 SCN8A L2HGDH ERI1 FTL HMG20A ZBTB33 ZNF592 EIF4G3 YIPF1 DALRD3 FLT1 MTOR THAP10 USP34 USP36 SMAD3 ATP6V0B MTMR6 KIAA1731 SFXN4 ZFP90 PDZD11 HSPB1 FAM3A RBM12B HIST1H3E ATXN7 MAD2L2 RCHY1 ADD3 SPIRE2 IGSF10 ANAPC16 ENSA ST20 C2orf79 DDX51 FIBP MYH9
Module 3 (brown)	TUBA3FP CNTN3 C9orf122 CCL11 MAGEL2 COMP PTPN5 PCDHA10 CXCL6 CCIN GP6 PCDHA4 ESRRB ILDR1 C21orf90 GLRB LINC02685 KNDC1 WIPF3 PDPN COL6A4P2 MAP1LC3C CDH19 RAB9B DHH C3orf36 FAM43B STMN2 PTGER4P2.CDK2AP2P2 WBSCR17 WDR17 DIO3 NBLA00301 CNTFR LRRC4C NALCN HAND2 EPHA10 CPZ SHANK1 KRT19 PCDP1 GRAMD1B CLDN4 SGCA ANXA8L2 GRM6 C5orf49 PTGFR MMP7 OGN BHLHE22 BICC1 CCBE1 TMEM90A ENPP5 TMEM132E PRSS8 DPT OMD SYT6 PRND SEZ6L2 GFPT2 NCAM2 ARMCX3 PLCXD3 SYT13 NTM GRAPL GRIP2 GDF6 RAD51AP2 MYOCD FAM160A1 PCDHB19P OPCML COL10A1 CCDC89 TMC5 AQP7P3 EDA CC2D2B MS4A2 CCL26 C8orf79 WNT4 CDS1 CPA3 RHBG HAS2 WFDC2 FAM180A POM121L9P TEKT3 DPEP1 ASPHD1 ZNF630 PI3 CASC15 FES MCTP2 H2BFXP SYNPO2L SAMD5 ITGB6 CRLF1 ZNF311 CSDC2 ACAN MEOX1 ST6GAL2 PRDM16 CILP2 FAP ARMCX6 BMPR1B CCDC80 PRKX GPR173 FAIM2 SHISA3 GSTM5 KIAA1244 PTGER1 BAI3 JPH1 NME5 LIPH BNC1 C1QTNF2 WISP2 CDSN KCNQ3 MOXD1 DARC RASL11B SAMD11 LRRC43 SNX32 PLEKHN1 IL17D VLDLR MFSD7 NKD2 SCG2 NUDT10 KCNIP3 BEND6 GLI2 C4orf31 FNDC1 MYOZ1 4-Mar PTPRS KCTD15 SHISA2 WSCD2 KIAA1644 SRPX HMGCLL1 CLEC4F KCNK3 PI16 SVEP1 SOX30 SOX8 FZD10 HAMP KIAA1211 CHRDL1 KCNA5 SGSM1 MFAP2 FAM101A ZDBF2 KCNF1 ARNT2 SULT1E1 HSPB8 TMEM200A SORCS2 LIMS3 ISLR SCN3B EMILIN1 CPXM2 PKP1 TOMM20L ULBP3 SLC34A1 COL8A1 RIPPLY1 ZNF287 SAMD12 TRPV4 GRID1 NBEA GPR143 KIAA1199 GLB1L2 FAM66C CACNA1H DKK2 ELOVL7 PBX1 GPC4 GAS1 SLC24A3 ZNF204P CLRN3 CRTAC1 BMX GPR77 CEACAM22P GPR182 COL4A4 PKNOX2 SPIRE1 SLC34A2 LPAR1 NPY5R KIAA1755 PTPN14 SCARA5 SLCO1C1 JAKMIP3 ANKRD1 ALDH1A3 PCDH7 C14orf37 COL6A6 KRT86 FLJ42875 REEP1 PCDHB18 C7 C21orf82 HUNK ZNF860 SSBP2 SSC5D COL16A1 FAM153A GLI1 MFAP4 TUB HTRA3 GALR3 SLC45A2 SDCBP2 MAP3K5 NEBL AEBP1 RAP1GAP2 SPON1 TMEM130 FGF18 TMC7 ANXA8 LY6H DES CD1D PRDM6 KRT81 NGFR POSTN MMP28 MYOM3 AMPH GLIS2 FAM196A ARHGAP10 GPBAR1 SGCD C1orf126 NAP1L3 CSMD2 PDE1A PCBP3 SYN1 C8orf48 EGFL6 PGR C10orf107 FAM190A RERGL EGR1 NTRK2 GPM6A COL8A2 TSPAN2 ULK2 RNF112 PAPLN C12orf68 CHST11 MFSD6 SNAI1 LINC00908 GJC2 LOC283174 GGN ADAMTS3 ARHGEF33 LAMA2 C13orf33 GREM1 LRRC45 ULBP1 FREM1 CPNE7 FOXL1 C2CD4A MMP23B LONRF2 VIPR2 NTF3 MRGPRF PRRT1 PCNXL2 ITGBL1 DACT3 FOXC2 PRSS35 ADRA2A COL13A1 C1QTNF7 PDZRN3 HRCT1 MUC13 C15orf39 NXN MMP16 THSD7B MAMLD1 PDZRN4 SFRP4 FAM19A5 ABI3BP FLRT2 LRRC10B COLEC10 PRELP SELP EFEMP1 MAP6 PODN CPXM1 LUM CHST7 RSPO3 REM1 CDHR3 TLX1 COL6A2 SLC26A10 DOC2B PLXNA4 CCL21 EGR2 F2RL2 RGS11 MGP SGK1 SULF1 GALNTL1 FIBIN ATP1A2 CRP PDGFRA ITGAV COL9A1 LRRC55 GGT5 NACAD CAPN6 WLS PCDHB14 LGALS3 EVC EML6 TMEM119 FAM171A2 FOSB DBC1 KCNS3 EFHB HSPB2 MXRA8 SULF2 MRC2 PDLIM4 PSORS1C1 NRXN3 PCYT1B CXCL14 INMT PRKD1 RCAN2 MN1 WDR86 GYPE ADCY5 RGS9 RSPH4A CTF1 C6orf165 CLEC11A AQP7P1 LOXL1 MYO7B CYP4B1 RGS17 STRA13 SPOCK1 CILP LPHN3 PTPRR ITGB8 TC2N OSR1 TLX1NB TNXB MB COL5A1 C11orf70 LEPREL2 CALHM2 PDZD4 NTRK3 FAM131B IGFBP7 PLN TAX1BP3 TSPAN11 SH3RF3 RSPH9 FAM124A CCDC74A LARP6 GPX7 KRT7 ARSI C8orf4 TIMP2 HMCN1 CYS1 VASH1 TTC39A SOD3 EIF4E3 FAM155A NPHP4 BGN ADAM15 CBFA2T3 C1orf70 PLEKHH2 KLF5 UNC5B SCUBE3 DCN PTPRT ROR2 METRNL ZNF518B C1orf198 PTH1R EFEMP2 SLC6A6 SUSD4 MYH11 ADAMTS6 FAM46B HSPB7 TIAM2 EPHA3 PTK7 SMARCD3 CACNA1C FOXF2 GULP1 FMOD VCAN MAB21L2

	STRA6 CHRDL2 GAS6 CSDAP1 PTGIS EFNB3 CERCAM ADAM33 FXYD6 SLCO3A1 PAPPA MMRN1 PTP4A3 C1QTNF1 CST2 CNFN EXOC3L2 KCNAB1 ENG HEYL ZDHHC15 DOK5 FAM227A TBXA2R FHOD3 LYPD5 C2orf40 ZNF135 CLIP2 CGB7 FOXS1 DSEL TWIST2 ADAM12 PTGIR CTHRC1 FA2H SGCB NFATC1 WISP1 FUT1 BRSK1 KLF8 PTPRZ1 RGS6 RECK TMEM51 OLFML1 STAT3 ANO4 MVK SUSD5 DNAJC18 PLA2G5 TMEM231 HOXB2 COL1A1 KCNK17 FMO2 BHLHE22.AS1 FMNL3 ITGA11 DLG3 ANGPTL1 ELN LTC4S IGSF22 PTGDS PTGS2 GPR1 SGIP1 EXTL1 GEFT C1orf203 GREB1 ADAMTS14 C9orf96 VTCN1 S1PR3 NPTX2 RCN3 ZFP36 ARHGAP17 3-Mar SMAD5OS VEPH1 C12orf53 DACT1 FHL2 HYAL3 ISLR2 DNAH2 CDH11 ODZ4 GPR124 AMOTL1 LTBP2 CCDC8 CRISPLD2 NAT6 CXCL12 DBN1 COL11A1 PIK3C2B FBLN2 RNF135 SYDE1 DPYSL4 RASAL1 MAB21L1 ADCY4 LRRC15 TNFRSF10C CRIP2 ASAM KRT80 FZD2 TPM4 RNF182 C8orf45 COL14A1 SEMA3C FSTL3 RASL10A FBLN5 SNCA SLC7A1 COL4A3 TIMP1 C17orf76 HES4 EPHB3 TNFAIP6 FCER1A MTMR9L LINC01144 LRRC4B PKHD1 S100A3 HCG11 CECR4 C10orf75 IL1RL1 LTK LINC02731 C15orf59 SMIM32 SDK1 BDKRB2 SLITRK4 NPDC1 VGLL3 DACH1 ANTXR1 CREB3L1 ARHGAP31 TACSTD2 LGR6 PODXL SLC39A10 IL3RA GPR162 PLXDC2 ZNF114 ALDH1A2 TBX1 C3orf52 C4orf49 NEGR1 ID4 MCC TMEM102 COL12A1 CD207 ECM1 RRAD MYH15 SEMA5A ZNF467 EBF4 PLAC9 HSD17B7 CTTNBP2 XKR6 C1orf204 KLF2 KAL1 F2RL3 PROCR TLR5 PMP22 PPP2R3A PRR16 MEIS3 CDKL2 TRPC4 KIAA1274 TCEAL7 S100A2 BMP8A KANK3 PPP1R14A THBD PCDHB12 NOVA2 DZIP1 HOXB4 NOS3 HOXA2 S100A6 SLC25A24 AFAP1 DGKE PRPH2 COL22A1 WTIP NRIP2 THBS1 NCOA7 FBN1 PRRX2 PODNL1 TCF21 DPYSL3 CD248 CEP68 MCF2L2 HIC1 CYP2D7P1 ZDHHC13 OSBPL5 C20orf194 HRH2 IQSEC2 5-Sep CAPN11 PPM1J ABR FZD8 EGFLAM ST3GAL3 TNN LDOC1L CLDN11 DCLK1 PELI1 SYT11 APBA2 CHD3 PRDM11 CHST6 NGF BEX4 SSPN TUSC3 C14orf129 JAG2 TPM1 CCDC48 TMCC3 TLL1 MEGF6 DUOX2 ADAMTS2 CD9 MAPK11 TCEAL5 CEND1 COL1A2 RYR2 SAMD14 ARHGAP24 CCBL1 MMP11 ST6GALNAC1 CCDC3 EHD2 LRRC70 TNFSF15 FAM57A KIAA1324L TEAD4 CRTC3 COL17A1 LGI2 RASL12 C20orf54 EMID1 ITGA10 LSAMP TGFA HSPG2 ANK3 FREM2 RNF150 PRICKLE1 CBX6 DYX1C1 ZNF608 TPSAB1 FOS CEBPA.DT LIMA1 EPHA4 TFPI2 NQO2 LAMC2 MAMDC2 FOXRED2 ZNF469 PNMA1 COL3A1 CD93 PNMA2 STK39 ZSCAN12P1 AHNAK2 C2orf55 ZNF853 OSBPL10 LOC100270710 LBH TMEM200C CCDC81 COL5A2 ACVR2B.AS1 PHYHIPL PABPC5 N4BP3 EPHB6 LPHN1 KIF26B USP43 LRRN3 LZTS1 MYOZ3 SFRP1 CRIP1 USHBP1 ABCG4 MSRB3 GSTA4
Module 4 (yellow)	CCL3L3 OMG MDS2 GPR141 DPRXP4 CEBPE C12orf70 BET3L C12orf63 TXNDC3 S100Z TSHR IL24 MCF2 TREML1 CCL24 MORN3 C1orf200 NTRK1 LINC01366 LOC100134868 ST6GALNAC5 KIR2DL3 CLEC12B SIX1 CATSPER1 ZNF80 LANCL3 CCR8 DNMT3L PCDH20 MUM1L1 PMCH KRT23 CDHR2 C14orf64 SLC12A3 BFSP2 SLAIN1 C20orf197 C14orf72 GAP43 EVC2 KIR3DL2 CD1B TCL1A CCR4 GGTLC1 RPH3A MTMR8 IL12B GPR63 POF1B C19orf77 NLRP12 HLA.DPB2 SPIC HTRA4 IL9R TMPRSS3 GSDMA RAB39 C4orf48 TRIM40 CACNA1F CLEC9A CXCL1 SIGLEC14 CADM3 CEACAM3 GPR84 TNFSF9 EMR4P INSL3 MANCR KHDC1 SLFN14 FOXD4L6 FUT8.AS1 GPR174 TNFRSF13C TSKS PLA2G2A RSPH1 WNT1 TMEM92 RIMBP3C C22orf34 SIGLEC12 GCNT3 LOC100240735 NELL2 C5orf30 LILRA3 C5orf46 CCL19 REN MSX2P1 CASP5 ERMN C9orf139 PBX4 KCNAB3 KLRC1 C20orf107 S100P FMN1 GPR82 IL7R PROK2 SH3D20 LMOD3 TNFRSF13B C9orf173 FLT3 C9orf109 CA14 ADAMDEC1 LGALS2 RNF175 LILRA1 B4GALNT1 CD1E KIAA1024 SCNN1B LMTK3 C9orf110 GCET2 CXCL13 GAPT LIMK2 CXorf65 SLCO5A1 ZNF683 WNT10B LY6E SLC24A4 RLTPR CAMK4 CD1A MGC29506 GPA33 LYPD1 ICOS KLHL3 PTCRA DOCK8 CLEC4E ARRDC5 OSBPL7 ARHGAP27P2 PAX5 PHOSPHO1 CEACAM4 ANKRD55 C19orf35 IL8 GPR55 CD38 MS4A1 CLECL1 SPINK1 TMEM106A ACR BLK NCRNA00092 C5orf20 GPR171 DTHD1 XCL1 ASNS CCL17 ARRDC3.AS1 CLEC5A WNT10A CD19 RHOF LINC02085 ZCCHC18 PLD4 AGPAT4 TNFSF8 CD1C LILRA4 DUSP26 PRAM1 HSH2D JAK3 CCL13 NAPSB CA12 PRKCQ C1orf170 C15orf48 LHFPL2 DNAJC5B SEMA4D PTPRO TNIK LINC00937 RGL4 SYCE1L DUOXA2 ALK

	GZMM ST8SIA1 RASA3 VENTX PRDM8 CD40LG ZC3H12D GGTLC2 P2RY12 GBP1P1 NOD2 SOD2 TMEM154 HPGDS OLR1 CHIC1 ZNF215 SLAMF1 ICAM1 GPR109B TOX LINC00654 THBS4 TLR2 SELM STYK1 APOBEC3H TRAT1 PGA3 STAP1 POU2AF1 TRIM22 EZR MTHFD2 NCR3 S1PR4 KCNA3 KLRF1 PLEKHO2 IGJ FBXO41 IL2RA ODF2L B3GALT4 CYTIP MPO P2RY10 CD3G PLA2G2D CD69 SLFN12L THEMIS P2RX4 PRDM12 FCRL5 IGLON5 LTA HAPLN3 ATP1B3 TIFAB FAM65B PTGES GHRL CKM MYO1G EMILIN2 SSTR1 TLR8 TNFRSF18 TBX21 C6orf105 PDE6G SIRPB2 CCR6 FPR2 CHIT1 NIPAL4 IFI27L2 C17orf39 GZMK RFPL1 KLHL6 TMEM144 PREX1 SIGLEC11 CCR2 IL21R C16orf54 ST6GALNAC4 C2orf27A CCL22 SPP1 TRIM6 HLA.DOB LY96 RASGEF1A ADAM6 GPR109A CD70 CD6 TLR10 TTYH3 FAM164A SIGLEC9 TIMD4 C6orf174 ABI3 MYL4 ETV7 LIF C20orf118 CASP1 CFP C11orf45 PPM1N PATL2 SPOCK2 MLC1 TRO CD300C LY9 APOBEC3D SH2D2A CXorf21 CARD9 SMTNL1 MARCO GRAP2 CXCL3 FPR1 ETV6 GJA3 IL23R SIGLEC10 TNFSF13B EGLN3 ACSL6 KCNH4 LCP1 S100B C4orf39 APOBEC3G PYCARD SYNGR3 TTC16 RAB39B CD2 TNIP3 DOK1 VNN2 COTL1 PDE4B GNB3 IFI16 VCAM1 MCOLN2 APOL3 TMC8 CD226 HK2 SLC1A7 C11orf21 ALOX5AP RAB33A PCED1B.AS1 MANBA TMC6 REC8 KLHL38 HTR7 RAB31 ZBTB32 ANO9 ZBED2 MX2 HLA.F SIGLEC5 C6orf150 TM6SF1 CIB2 IL10 GAS7 PTGER4 BIRC7 SLFN12 TLR7 SGTB SYK APOBEC3A ALOX5 SLC4A10 SIT1 DDX26B CNR2 GLDN SIGLEC7 ITK PKIB C7orf23 TMIGD2 ICAM4 PPP1R3G PHLDA2 GGT3P AREG SEMA3D MYO5A CD84 SLC1A5 C7orf11 ZBP1 IL18RAP CD80 C20orf132 SIGLEC8 FAM176B BTLA SLC7A7 TAGAP BCL11A RGS2 FCGR1A LINC00426 CYBB CIITA C5AR1 SPIB NCF1B BCL11B FYB ANK2 ADD2 GPR68 ABCC1 ACSS1 CD27 C1orf59 LEFTY1 GLIPR2 RBM3 CXCR5 SULT1C2 DGKG TMEM149 RBM45 TFEC LGALS9 ITGA4 LIMD2 CCL18 LILRB3 SIGLECP3 GNB4 TNFRSF11B ATP6V0E2 IL27RA CCL20 TPK1 GIMAP5 NUDT8 DAPP1 DUSP5 AXL TMED3 GALNT12 MSR1 HMHA1 PSTPIP1 IMPDH1 CYTL1 ADAM28 ICAM5 RAB3IL1 MCART6 MYO1F ZSCAN21 FAM35B2 SYTL1 GPR132 GATA3 MAL EHD1 KCNK6 CYP4F35P NNMT RHOH WDR1 BCL2L14 LOC154761 LY75 ARPC1B HLA.DQB2 CD5 JAKMIP1 BCORL2 ARHGAP4 HCLS1 HLA.DMB CMKLR1 KLRC2 YPEL4 GIMAP4 RMND5A LILRB4 ZNF831 CARD16 CD97 FCHSD1 SRGN BANK1 GZMA PRKCA TMEM173 TRPV1 AKAP1 MNDA RASAL3 P2RY8 C5orf56 GPR114 KIAA1377 CCL3L1 CLEC10A KRT17 SNAI3 GAL3ST4 HPSE CSDA SH3BP1 ANXA2P1 FAM166A RPS6KA1 TMEM229B ABCG1 THAP7.AS1 FAM27C TNF FAM115C CYP2S1 ARHGAP30 PDE4A CDH3 FCGR1C BCL3 C16orf57 DPEP2 KCTD12 KCNQ5 IFNAR2 SAMSN1 HDDC2 KLRC3 PFN2 CD68 KIAA0748 PEX11B DOCK11 RGS18 PPP4R1L LAPTM5 BIRC3 PLTP LEF1 CCDC136 CXCR2P1 SLA2 FLI1 MTMR15 MEI1 HIST1H2BD FUT7 PLAU CSF2RA ITGAM SAMD9L MS4A14 MYBPC3 TCTA CD79A C3AR1 ARHGAP15 KLRB1 MSN FCRL3 DENND3 CD44 CTLA4 RCSD1 SLA LOC606724 NCCRP1 TBC1D10C LINC02591 NFKBID OSTF1 SKAP1 RUNX3 B3GNT4 HAVCR2 LTB LOC644936 CYTH4 FYN TRPS1 TLR6 TRAF5 CPVL HLA.DMA TMEM55A SLC25A12 SCML4 NOTUM CD3E SP140 FCHO1 MLLT3 GP1BA DOK2 ETV5 CARD6 TXNDC9 SAMD3 MPP3 OSGIN2 LRRK1 ADAP2 KIAA1949 CLEC7A LILRA6 EVI2A ITGAD CD40 SLAMF6 GBP6 RPP25 NFKB2 STAT4 TNFRSF8 ARHGAP27 PAG1 HAR1A LCP2 GNA15 RNASE6 TRAF3IP3 ABCD2 LINC02447 CR1 ACAP1 TGM1 C1orf38 TLR1 FSD1 NCF1C OSCAR ATP6V0D2 SOCS1 CCR7 FCGR3A BTG1 MMP9 CAPG SLC2A6 ARL14 PTGDR SEMA6C FUT8 CD48 RIMBP3 GPR160 HOMER1 PRKD2 AIM2 GLTP GIMAP8 P2RY13 CCL2 CD22 NCKAP1L SLC4A8 AMPD3 DUSP2 LYL1 PTPRC HCG26 PIK3R5 CD4 APH1B NCRNA00173 CD247 CCR5 PDCD1LG2 SECTM1 CECR1 LILRA5 WDFY4 C13orf18 TNFRSF9 RASGRP1 TNFRSF1B TRIM58 CMTM3 STX11 FASLG NCR1 TXK FERMT3 SLC9A9 TNFAIP8 VMO1 GMIP POU3F1 HLA.G EID3 SBNO2 DOCK10 SEPW1 IRF1 HK3 LAIR2 MSH3 ANKRD22 C19orf38 BTK FNBP1 GPR172B SAP30L ARHGDIB LRRC33 P2RX1 PTPLAD2 UBASH3A NCF2 MAPK3 IDO1 SERTAD3 C1orf162 LILRA2 RASGRP4 NFS1 SH3BGRL CBARA1 FAM27A FBXW7 SELPLG FAM179A CMTM7 PYHIN1 PAXIP1.DT PADI2 NBEAL2 DHRS9 NLRP3 CCL3 HLA.H PTGER2 LAIR1 CD300LB NFAM1

Module 5 (green)	ASXL3 FAM3B EVPLL CPLX2 CYP2A13 HSD17B13 FAM99B CNDP1 SUGT1P1 PRAMEF8 ANKRD35 SLC5A11 BREA2 CYP2A7 ACTG1P4 C3orf32 DCAF13P3 PRAMEF10 ATF5 SLC26A7 FERMT1 RPL36A DAB1 MRAP2 MFSD2A MGC27382 CLCN1 BBOX1 IGF1 FNDC5 GPR88 CABYR IGFALS TSPO2 UGDH YWHAZ TKT PLAG1 TRIM16L FAM198A ZNF706 NSMCE2 ASPA DPF3 DCAF13 ZNF696 INTS8 MGC42105 ARPP21 LYZ CHRAC1 ACACB ATP6V1C1 TMEM249 FBXW10 MAPK8IP1P2 EIF3E ZNF7 SLC25A34 LCN2 MAF1 RELN FAM91A1 JRK NIP7 RPL30 SPTBN4 FAM134B UTP23 ZNF707 COMMD5 SQSTM1 GRIN2C SRXN1 AMY1A PABPC1 MTDH PHF20L1 C20orf3 TOP1MT ZNF596 MKI67IP C8orf77 CDA C8orf59 NUDCD1 STK3 PUF60 C8orf30A CPSF1 AMY2A SHARPIN GPR172A PPP1R3B ZNF16 ZC3H3 PVT1 EPS8L3 TCEB1 MTERFD1 RUNDC2C C8orf76 HSF1 LRRC69 ZNF250 ZFP41 RABGGTB RSL1D1 ENY2 RRS1 KCNE1 TTC35 WDYHV1 ACSL3 HSD3B2 C8orf33 RPL8 GARNL3 UBR5 DRAIC AMY2B TSNARE1 PRODH ZNF517 GALC MAPK8IP1 NME1.NME2 FKBP4 USP2 NOB1 GRAMD1C RARA STAU2 EXT1 TMEM68 HSD3B1 SLC23A3 C8orf38 CHCHD7 LARS
Module 6 (red)	ZNF660 ZNF471 ZNF665 ZNF391 ZNF14 ZNF813 ZNF83 ZSCAN18 ZNF229 ZNF285 ZNF681 ZNF793 ZNF85 ZNF714 ZNF43 ZNF502 ZNF320 ZNF528 ZNF382 ZNF577 VN1R1 ZFP82 ZNF667 ZNF389 ZNF137 ZNF835 ZNF549 ZNF167 ZNF431 ZNF418 ZNF404 ZFP28 ZNF607 ZNF816A ZNF321 ZNF701 ZNF732 ZNF486 ZNF347 ZNF880 KPNA5 ZNF256 ZNF501 ZNF737
Module 7 (black)	NAMPT BAT4 CSNK2B TAF11 TJAP1 CCHCR1 LEMD2 TNFSF14 C6orf153 EHMT2 CUL9 NOL7 TOMM6 RPP21 PEX3 YY1AP1 MEA1 RSL24D1 PFDN6 NCRNA00219 KLHDC3 GNL1 RING1 HSD17B12 TMEM14B SRF ZBTB22 SKIV2L C6orf48 VARS GAS5 PHF1 CA13 BAT3 TRIM27 PPP2R5D MTCH1 ZNRD1 SNRPC TBCC BYSL LYRM4
Module 8 (pink)	C1QBP GLOD4 SPAG7 RABEP1 MED11 SCO1 MPDU1 ELAC2 XK RNMTL1 EIF4A1 NEURL4 MED31 PAFAH1B1 C17orf85 MAP2K4 ALKBH5 MED9 RNF167 YWHAE FXR2 RPAIN SMCR7 DHRS7B UBE2G1 EIF5AL1 KIF1C ANKFY1 NUP88 ATPAF2 DRG2 ZSWIM7 CAMTA2 TSR1 LRRC48 WDR81 TMEM93 KIAA0664 INPP5K ZZEF1
